# Book Review

**DOI:** 10.1155/S0962935196000348

**Published:** 1996-06

**Authors:** Huub F. J. Savelkoul

**Affiliations:** 1 Department of Immunology Erasmus University Rotterdam The Netherlands


Mediators of Inflammation, **5**, 235–236 (1996)

### Book Review

Centner J, De Weck AL. *Atlas of Immuno-Allergology, An Illustrated Primer for Health Care Professionals. 3rd ed.* Gottingen: Hogrefe & Huber Publ, 1995.

This book is intended to create a better understanding of the complex principles and potential applications in the field of clinical immunology and allergology for health care professionals. Moreover, this book can set the stage for a better understanding of the practical immunological principles used in immune pathology. This 186 page atlas is well illustrated and divided into five sections dealing with fundamental mechanisms in immunology, common allergic diseases, elements of immune pathology, allergens and vaccines and immunological tests *in vitro*.

To explain the general concepts in immunology in an understandable way is not easy. Also this book did not completely succeed in this task. The potential use of an atlas like this is certainly dependent on extensive cross-referencing that is, unfortunately, not consistently present. The book lacks an extensive index as well. In some parts the abbreviations and the numbering are not explained or only explained in later sections without referring to that section. In the descriptive part on the immune system more quantitative information on the relative abundance of the various cell types and molecules (e.g. antibodies) described would be helpful. Moreover, at several places small typing errors or the omission to explain several abbreviations confuse the reader. In the third edition of this book such errors should have been removed. Essential to the understanding of the complex interactions between cells and molecules of the immune system is the histology of the lymphoid organs (Sections 1.4 through 1.6). This part of the book is clearly written and well illustrated. A point of criticism is that when describing lymphocyte recirculation the presence of different vascular addressins and high endothelial venules deserves to be discussed.

Some of the concepts presented clearly have to be updated according to current thinking. The role of co-stimulatory molecules (CD28) and their interaction with B-7 molecules have to be dealt with in relation to the section on T cell anergy induction where these molecules are not mentioned. Lately, the role of the dendritic cell as an important primer in the generation of immune responses has been appreciated and should be mentioned. Also in the section of cytokines it would be advisable to describe the concepts of pleiotropy, redundancy and receptor-mediated activity, that are so characteristic of all cytokines by definition. In the section on antibodies some quantitative information could be useful to illustrate the overwhelming degree of specificity in the humoral part of the immune system. The concepts of idiotypic and isotypic markers are introduced but not explained structurally.

Well explained for health care workers are the parts of the book dealing with immuno-allergic reactions, allergic diseases and the section on allergies and vaccines. Evidence has been provided recently that suggests that one or more susceptibility genes are localized on chromosome 5q31–q33 that are important to the development of asthma. These genes appear important in the genetic regulation of the inflammatory phenotype of asthmatics, like total IgE production (IL-4, IL-13, IL-12), eosinophilia (IL-5), growth factors (IL-3, IL-9, GM-CSF), growth factor receptors (CSF-1R) and adrenergic receptors (beta-2 and alpha-1B). A hallmark of atopy, however, is the presence of antigen-specific IgE antibodies in the serum. Especially here, several typographical errors confuse the reader. In the description of allergic diseases the potentially important role of chemokines for the attraction of eosinophils has not been described. The eosinophil is generally held responsible for the tissue damage that can be observed in the lungs of asthmatic patients. Its description only at the start of the book seems to be out of place. The rationally designed new drugs blocking the

© 1996 Rapid Science Publishers recruitment and activation status of eosinophils could have been described to further illustrate the important concepts that have been developed over the last few years with respect to the mechanisms of these allergic diseases. The mode of action of corticosteroids, the specific down-regulation of cytokine gene expression, is illustrative of the regulatory potential of several cytokines, like IL-4 and IL-5, in allergy and asthma.

The part on immune pathology contains an arbitrary selection of diseases in which the immune system is involved. Some examples are well explained and contribute to further understanding of fundamental concepts in immunology, such as the section on autoimmune diseases and the section on transplant rejection and HLA antigens. Other sections, such as that on HIV infection and loss of T cell responses, renal immunopathology and GVH seem to contribute less to the general understanding. The description of the antinuclear antibodies belongs in the technical part of the book. The section on immunosuppressive therapies lacks a schematic representation of the inhibition of IL-2 synthesis by T cells, since this mechanism is probably the best example of how an agent can be effective *in vivo* by inhibiting cytokine gene expression.

Part four of the book is composed of several different subjects primarily dealing with immunotherapy in general and hyposensitization and vaccination in particular. The section on immunotherapy of cancer is one of the best sections in the book with respect to the realistic illustrations employed. The accompanying text, however, is very concise. This section certainly illustrates the use of important basic concepts in immunology to the use of designing immunotherapy protocols in a rational way. It is rather surprising to repeat in this atlas for 'health care professionals' the principle of vaccination by recombinant vaccines in fish

Another very useful feature of this book is the section of immunological tests. By the use of coloured schematic illustrations in combination with concise technical descriptions the essential background of various common immunological techniques is well explained. The authors have achieved this complicated task by describing in an accessible way the very basics of many techniques currently employed routinely in many research and clinical chemistry laboratories. The highly informative and lifelike drawings have certainly added to the easy understanding of the sometimes complicated concepts on which these methods are based.

In conclusion, when explaining the important concepts of immunology and allergology to novices in the field it is important to provide a clearly written and concise text accompanied by carefully selected and appealing drawings. This atlas in certain sections certainly meets these criteria. Unfortunately, even this third edition still contains too many errors and omissions, and lacks extensive cross-referencing to facilitate its full use. Overall, this atlas offers a combination of introductory information into important concepts in immunology with insights into basic aspects of certain immunopathological diseases. Combined with some practical information on the application of major immunological techniques in such a comprehensive fashion this atlas is advisable to novices in the field or those that seek a quick reference to immunology and allergology.

**Huub F. J. Savelkoul**

Department of Immunology, Erasmus University, Rotterdam, the Netherlands

